# Comparison in dietary patterns derived for the Canadian Newfoundland and Labrador population through two time-separated studies

**DOI:** 10.1186/s12937-015-0064-6

**Published:** 2015-08-01

**Authors:** Zhi Chen, Peizhong Peter Wang, Lian Shi, Yun Zhu, Lin Liu, Zhiwei Gao, Janine Woodrow, Barbara Roebothan

**Affiliations:** 1 Division of Community Health and Humanities, Faculty of Medicine, Memorial University of Newfoundland, St. John’s, NL A1B 3V6 Canada; 2 Clinical Epidemiology Unit, Faculty of Medicine, Memorial University of Newfoundland, St John’s, NL Canada

**Keywords:** Dietary habits, Dietary patterns, Nutrition epidemiology, Newfoundland and Labrador population

## Abstract

**Background:**

While a dietary pattern is often believed to be stable in a population, there is limited research assessing its stability over time. The objective of this study is to explore and compare major dietary patterns derived for the Canadian subpopulation residing in Newfoundland and Labrador (NL), through two time-separated studies using an identical method.

**Methods:**

In this study, we derived and compared the major dietary patterns derived from two independent studies in the NL adult population. The first study was based on the healthy controls from a large population-based case–control study (CCS) in 2005. The second was from a food-frequency questionnaire validation project (FFQVP) conducted in 2012. In both studies, participants were recruited in the same manner and dietary information was collected by an identical self-administered food-frequency questionnaire (FFQ). Exploratory common factor analysis was conducted to identify major dietary patterns. A comparison was conducted between the two study populations.

**Results:**

Four major dietary patterns were identified: Meat, Vegetables/fruits, Fish, and Grains explaining 22 %, 20 %, 12 % and 9 % variance respectively, with a total variance of 63 %. Three major dietary patterns were derived for the controls of the CCS: Meat, Plant-based diet, and Fish explaining 24 %, 20 %, and 10 % variance respectively, with a total variance of 54 %. As the Plant-based diet pattern derived for the CCS was a combination of the Vegetables/fruits and Grains patterns derived for the FFQVP, no considerable difference in dietary patterns was found between the two studies.

**Conclusion:**

A comparison between two time-separated studies suggests that dietary patterns of the NL adult population have remained reasonably stable over almost a decade.

## Introduction

Most nutritional epidemiological literature addresses the use of nutrients or individual food items to assess possible associations between diet and health. There are several limitations of the single-nutrient approach: people eat meals consisting of a variety of foods rather than isolated nutrients; single-nutrient analysis does not account for complicated interactions among nutrients [1]; and nutrient effects are difficult to examine individually by single-nutrient analysis due in part to high levels of nutrient-nutrient interactions (for example, potassium and magnesium) [2]. Moreover, single-nutrient analysis may be confounded by each individual’s dietary pattern which is commonly associated with nutrient intakes [3, 4]. In order to overcome these limitations, an emerging approach in nutritional epidemiology is to use dietary patterns rather than isolated nutrients. Compared to the traditional approaches used in previous nutritional epidemiology, dietary pattern analysis considers how food and nutrients are consumed in combination and could therefore provide a more accurate and comprehensive description of dietary exposure in a certain population [4–6].

Support for the use of dietary pattern analysis has been growing. People’s eating habits usually remain relatively stable unless they experience such major changes in their personal circumstances as getting married, changing geographic location or receiving a serious warning from a health professional that their current diets have significant and negative impacts on their health. Many factors influence food choice including family income, food prices, individual preferences and beliefs, cultural traditions and customs, as well as geographic and environmental factors [7, 8]. While a dietary pattern is often believed to be relatively stable in a population, limited research has assessed its degree of stability over time. The objectives of this study were 1) to identify the major dietary patterns of an NL population from two time-separated studies using identical methods and 2) to explore whether there were differences in these dietary patterns between these two studies conducted several years apart.

Our large and multidisciplinary research team, including more than 40 researchers residing in the provinces of Ontario (ON) and NL, Canada, has published several journal articles on diet of the NL adult population using both nutrient and dietary pattern methods [9–13]. Using exploratory common factor analysis, this study derived and compared major dietary patterns from dietary data collected by use of a food-frequency questionnaire (FFQ) in two projects conducted with the NL population, a population-based case–control study (CCS), 2001 – 2005 [14, 15], and a food-frequency questionnaire validation project (FFQVP), 2012 [16].

## Methods

### Study participants

The CCS was conducted from 2001 to 2005 and a detailed description of selecting the population controls can be found elsewhere [17]. Briefly, eligible cases were newly diagnosed colorectal cancer patients. Controls were frequency-matched with cases by sex and age on 5-year strata. Both cases and controls were selected from NL residents, aged from 20 to 74 years. They were identified through random digit dialing using phone numbers provided by a NL phone company (Aliant). By July 2005, a total of 2168 eligible controls were contacted for further survey and 1603 controls agreed to participate. Those persons who agreed to participate were sent a survey package, comprised of a written consent form, a personal history questionnaire (PHQ), and a food-frequency questionnaire (FFQ). Of these, 717 participants completed and returned the survey package with a response rate of 44.7 %. Current study participants were part of the population controls from the CCS project, aged from 35 to 70 years.

The FFQVP was conducted between February 2011 and May 2012, by the Health Research Unit of Memorial University. This study population was sampled by stratified random digit dialing with proportional allocation methodology. Participants were recruited in the same manner as was used by the CCS. All were non-institutionalized adult residents of NL, aged 35–70 years. Residence in NL was defined as having lived in the province for at least two years prior to the beginning of the study. Other inclusion criteria included a minimum of an 8^th^ grade level of English speaking and reading skills and no cognitive impairment, psychological conditions, or pregnancy. From a list of phone numbers (landlines and cell phones) provided by Info Canada, an initial sample of 450 persons was recruited randomly by telephone. After exclusion, 306 participants were identified as potential respondents and mailed a survey package containing a written consent form and a FFQ. Finally, 205 individuals completed and returned the survey package, giving a response rate of 67 %.

**Fig. 1:**
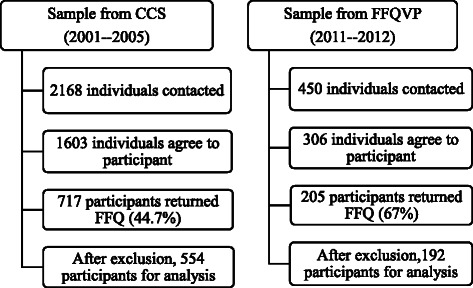
Participant recruitment for FFQVP and CCS

Data from FFQs with 20 continuous blanks or reporting energy intakes outside the range of 500–5000 kcal were excluded [18]. After exclusion, a total of 554 participants of the former population and 192 participants of the current population remained and data provided by them were entered into further analysis (Fig. 1).

### Data collection

For both studies, a modified FFQ, based on the well-known Hawaii FFQ, was used to gather dietary intake data. The original Hawaii FFQ was used in Hawaii/Southern California to assess the general food intake of a multi-ethnic population [19]. It has been validated and widely used in the United States [20–22]. In our new version of the FFQ, some food items unusual to the NL population (for example, tamales and ham hocks) were deleted or altered, and some items commonly consumed in NL (for example, cloudberries/bake-apples, game meats, and pickled/smoked fish) were added. The food items listed in the NL FFQ, which has been validated by our team [16], include nine major categories: beverages, dairy products, mixed dishes, vegetables, meat and fish, cereals and grains, fruits, desserts and sweets, and miscellaneous.

Dietary assessments of participants using data collected via this FFQ were carried out 12–24 months prior to conducting a telephone interview. During the interview each participant was required to recall food intake over the past 24 months - the frequency of each food item consumed, the number of servings, and the approximate size of portions habitually consumed at a single sitting. The units of frequency ranged from per day, per week, per month to rarely or never, and the portion sizes included standard and smaller or larger than standard (standard ± 25 %). A standard serving size for each food item or beverage was described on the FFQ in common household measures and grams. The FFQ also included food photographs to indicate standard portion sizes.

Except for several independent food items, the 169 food items were categorized into 39 predefined food groups, based on their nutritional characteristics and their role in the diet (Table 1). Independent food items comprised their own groups, given that they could not be appropriately combined with others, for example, eggs, beer, jam, and fruit pies. Total energy and nutrient intakes for individuals were calculated according to the composition values from the 2005 Canadian Nutrient File (CNF) and the Elizabeth Stewart Hands and Associations (ESHA) Food Processor database software [23].

**Table 1:** Food groups used in the dietary pattern analysis

Food group	Food items
Milk	Whole milk, 2 % milk, skim milk, milk shake
Yogurt	Yogurt drink, yogurt (regular/light, plain/fruit/frozen)
Coffee	Coffee (regular/decaffeinated)
Tea	Tea (regular/herbal)
Sugar	Sugar (in tea/coffee)
Soft drinks	Cola, Pepsi, diet/other soft drinks
Egg	Egg (boiled/fried)
Cheese	Cream cheese (regular fat), cheese (regular fat, light, ultra light), cottage, ricotta cheese
Mixed dishes	Soups (creamed), pasta (with meat sauce), mixed dishes (with cheese), pizza (with meat), meat stew with vegetables, chili with meat
Red meat	Ground beef (regular/medium/lean), roast beef, steak, pork chop, roast pork, baked ham, bacon, veal, lamb, hot dog, wiener, sausage, corned beef, cold cuts
Game	Sea-bird, seal, caribou, moose, partridge, other wild birds
Cured/processed red meat	Baked ham, bacon, hot dog, wiener, corned beef, cold cuts, salted/dried meat, pickled meat
Cured/processed meat	Baked ham, bacon, hot dog, wiener, corned beef, cold cuts, fried chicken, salted/dried meat, pickled meat, fried/canned/smoked/salted/dried/pickled fish
Poultry	Fried chicken, chicken (roasted or stewed/skin removed)
Fish	Shellfish, fish (baked or broiled), fried/canned/smoked/salted/dried/pickled fish
Processed fish	Canned/smoked/salted/dried/pickled fish
Fruit juice	Orange/grapefruit/apple/grape/other fruit juice, fruit drinks/lemonade, iced tea
Other fruit	Apple, pear, grape, banana, beach, plum, nectarine, apricot, cantaloupe, watermelon, honeydew melon, mango, papaya, apple sauce, all other fruit with the exception of berries
Root vegetables	Potatoes (mashed, baked), fried potatoes/French fries, carrots (raw or cooked)
Cruciferous vegetables	Broccoli, cabbage, coleslaw, cauliflower, asparagus or brussel sprouts
Other green	Spinach/other green-leaf vegetables, green salad
Beans, peas	Peas, lima beans, green/yellow beans, beans/lentils, pea soup
Tomato sauce	Tomatoes (fresh/canned), ketchup
Other vegetables	Corn, cucumber, onions, beets, yellow squash, zucchini or eggplant, sweet pepper, bean sprout, avocado, other vegetables
Total cereals and grains	Bran or granola cereal, whole wheat cereals, cereals (not sugar coated), hot cereals, sugar coated cereals, other breakfast cereals, sugar on cereal, 100 % whole grain/dark bread, 60 % whole grain/light rye, white bread, white bread rolls, whole wheat rolls, crackers, bran/oat muffin, other muffins, pancake, waffles, macaroni, spaghetti, noodles, rice, crisp snacks
Whole grains	Whole wheat cereals, 100 % whole grain/dark bread, 60 % whole/light rye, whole wheat rolls
Dessert and sweet	Cakes, pies and tarts, donuts and sweet rolls, cookies, iced cream, light or diet ice cream, pudding, diet or light pudding, jell-o, popsicles, freezies, candy (with/without chocolate)
Vegetable juice	Vegetable juice
Beer	Beer, ale
Whiter wine	White wine
Red wine	Red wine, sherry
Liquor	Liquor
Citrus	Citrus fruits
Berries	Berries
Dried fruits	Dried fruits
Canned fruits	Canned fruits
Pies, tarts	Pies, tarts
Jam, jelly	Jam, jelly, honey syrup
Pickled vegetables	Pickles, relish

The study sample from the CCS was administered a PHQ to collect socio-demographic and medical information including age, sex, date of birth, marital status, educational attainment, medical history (for example, history of diabetes or high cholesterol), bowel screening history, medication use (for example, multivitamins and nonsteroidal anti-inflammatory drugs), alcohol and tobacco use, reproductive factors, self-reported physical activity and other information.

Less extensive socio-demographic information was gathered as part of the telephone interview with the study population in FFQVP. This included age, sex, size of community, marital status, employment status, level of education, and smoking habits.

### Statistical analyses

The appropriateness of factor analysis for each study sample was verified by Bartlett’s Test of Sphericity (BTS) and the Kaiser-Meyer-Olkin (KMO) measurement. BTS was used to test the homogeneity of variances and KMO measurement was conducted for testing sampling adequacy. KMO values could not be less than 0.5 to ensure the suitability of factor analysis use in this study [24]. Exploratory common factor analysis was used for factor extraction, and orthogonal rotation (varimax option) was used for simpler structures with greater interpretability. A factor was retained when it met the following criteria: factor eigen value > 1.50, identification of a break point in the scree plot (the difference between each two points becomes small suddenly), the proportion of variance explained (at least 50 % of variance in this study), and factor interpretability (the fewer the factors, the greater the interpretability). A rotated loading matrix described the strength and direction of the associations between the retained factors and food groups. If a food group had a factor loading ≥0.5 (for the FFQVP population) or ≥0.35 (for the CCS population), it was loaded on a factor. We also retained food groups that had negative correlations (≤ − 0.2) to incorporate the valuable information concerning infrequently consumed foods within each factor [25]. Dietary patterns were named according to the characteristics of food groups loaded on a retained factor.

Differences in demographic information between the two study populations were detected by *t*-test and chi-square test. Statistical analyses were performed using the Statistical Analysis System (SAS, version 9.2) software. Differences with *p*-value <0.05 were considered to be statistically significant.

### Ethics statement

This research was approved by the HREB at Memorial University of Newfoundland. (Reference number 14.098).

## Results

### Demographic information

In total, the study sample was made up of 554 participants from the CCS population and 192 participants from the FFQVP population. All of the study participants were aged 35–70 years. Individuals from the CCS (58.7 ± 7.7) were significantly older than those from the FFQVP (56.2 ± 8.7). The gender distributions between the two populations were significantly different (*p* < 0.0001). The percentage of males in the CCS (58.1 %) was much higher than in the FFQVP study (22.4 %). Also, distributions of education attainment and marital status between these two study groups were significantly different (Table 2).

**Table 2:** Demographic information of study participants from both CCS and FFQVP

Demographic information	CSS	FFQVP	P^a^
Age (mean ± SD)	58.7 ± 7.7	56.2 ± 8.7	<0.0001
Sex			<0.0001
Male	322 (58.1 %)	43 (22.4 %)	
Female	232 (41.9 %)	149 (77.6 %)	
Marital Status			<0.0001
Single	17 (2.9 %)	15 (7.8 %)	
Separated/divorced/widowed	74 (13.4 %)	26 (13.5 %)	
Married/living together	463 (83.7 %)	151 (78.7 %)	
Level of education			<0.0001
Some school without high school certificate	156 (28.4 %)	27 (14.0 %)	
High school certificate	300 (54.6 %)	51 (26.6 %)	
Post-secondary education	98 (17 %)	114 (59.4 %)	

^a^*P* value from *t* test within CCS and FFQVP groups

### Factor analysis

**Fig. 2:**
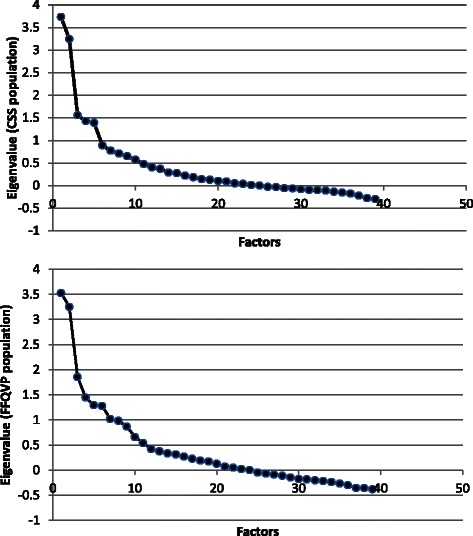
Scree plots for eigenvalues from factor extraction in two studies

The observed KMOs for the two populations were 0.68 for the CCS and 0.60 for the FFQVP suggesting that the two samples from different populations were adequate for factor analysis. *P* values from the BTS were <0.0001, suggesting homogeneity of variance across the samples. Figure 2 shows the scree plots for both study populations. For the CCS sample, the first three eigenvalues, 3.73, 3.24, and 1.56, drop substantially. After the fourth eigenvalue (1.43), the values remain more consistent (1.39 for the fifth, and 0.89 for the sixth). As a result, the third point is considered a break point. As for the FFQVP sample, differences between each two eigenvalues change to gentle from sharp after the fourth value. Accordingly, the fourth point is regarded as a break point on this plot. All eigenvalues before each break point are greater than 1.50. Combined with total variance explained and factor interpretability, a 3-factor solution was selected for the study population from CCS. This explained 54 % of variance. The first four factors were retained for the study population from FFQVP, and this explained 63 % of variance (Table 3).

**Table 3:** Factor Loadings and Explained Variances (VAR) of the Major Dietary Patterns identified in two studies, using an exploratory common factor analysis

Food groups	Factor loadings^a^						
	Current population				Former population		
	Meat	Vegetables/Fruits	Fish	Grain	Meat	Plant-based diet	Fish
Milk							
Yogurt							
Coffee				−0.31			
Tea							
Sugar							
Soft drinks				−0.20			
Egg							
Cheese							−0.24
Mixed dishes					0.43		
Red meat	0.83				0.88		
Game							
Cured/processed red meat	0.90				0.91		
Cured/processed meat	0.93				0.92		
Poultry							
Fish			0.78				0.73
Processed fish			0.70				0.68
Fruit juice		−0.25					
Other fruits						0.42	0.48
Root vegetables							
Cruciferous vegetables						0.58	
Other greens		0.68				0.50	
Beans, peas						0.52	
Tomato sauce		0.60				0.41	
Other vegetables		0.75				0.57	
Total cereals and grains				0.55		0.38	
Whole grains				0.52		0.36	
Desserts and sweets							
Vegetable juice							
Beer				−0.24			
White wine				−0.26			
Red wine							
Liquor							
Citrus							
Berries		0.50					0.49
Dried fruit							
Canned fruit							
Pies, tarts							
Jam, jelly							
Pickled vegetables							
Proportion of VAR explained (%)	22 %	20 %	12 %	9 %	24 %	20 %	10 %
Cumulative VAR explained (%)	22 %	42 %	54 %	63 %	24 %	44 %	54 %

^a^Factor loadings ≥ 0.5 will be loaded on a factor in FFQVP population while factor loadings ≥ 0.35 will be loaded on a factor in CCS population; negative loading ≤ −0.20 will be retained; other loadings are not shown in the table

According to the results obtained from the factor loading matrix shown in Table 3, the retained factors were labelled, depending on the given food groups loaded on them. A factor loading ≥ 0.35 of a certain food group indicated a greater contribution of that food group to the specific pattern for the CCS population. The three retained factors were identified as three dietary patterns and were labelled Meat, Plant-based diet, and Fish. The first pattern was defined as the Meat pattern, and characterized by high loadings for red meat, cured/processed red meat, cured/processed meat, and mixed dishes. The second pattern, which loaded heavily on fruits, cruciferous vegetables, other green vegetables, beans, peas, other vegetables, tomato sauce, total cereals and grains, and whole grains, was labelled the Plant-based diet pattern. The final pattern was named Fish because it had high loadings of fish, processed fish, berries and other local fruits and negative loadings in the food groups of cheese.

The four retained factors were identified as four dietary patterns for the FFQVP population and were labelled Meat, Vegetables/fruits, Fish, and Grains. The four-factor dietary pattern was identified based on the results retained from the factor loading matrix (Table 3), where a higher factor loading of a given food group indicated a greater contribution of that food group to the specific pattern. The first pattern was labelled because of a high intake of red meat, cured/processed meat, and cured/processed red meat. The Vegetables/fruits pattern indicates a preference for several vegetable/fruit groups, including greens, tomato sauce, berries, and other vegetables. The Fish pattern had an emphasis on fish and processed fish. We named the final pattern Grains, since it was characterized by a high consumption of whole grains, cereals, and grains, and a low consumption of beer, white wine, and coffee.

## Discussion

Even though dietary pattern analysis has emerged as a possible approach examining possible diet-health relationship, little research has been conducted to assess the stability of dietary patterns derived for an identical population over time. In this study, we compared the major dietary patterns derived from two time-separated studies of the NL adult population assessed by a self-administered comprehensive FFQ.

The present study derived a three-factor dietary pattern for the CCS and a four-factor dietary pattern for the FFQVP. We observed both similarities and differences in dietary patterns between the two studies. The total variances explained for the CCS and FFQVP studies were similar, 54 % and 63 %, respectively. Both identified meat and plant-based food as the top two major factors, which in combination explained almost equal amounts of variation (42 % and 44 %). According to the factor loading matrix, the patterns labelled Meat pattern and Fish pattern derived for the CCS were largely the same as those two derived from the FFQVP. The meat pattern was similar to the Western pattern of many previous studies [26, 27] in the food items contained (for example, red meat, processed meat, other high-fat food). This pattern has been positively associated with cancer [28], cardiovascular diseases [29, 30], and obesity [31]. The Fish pattern, which is characterized by high intakes of fish and processed fish, seems to be different from any pattern described in other research. Geographic isolation and the historical importance of the cod fishery in NL may be the leading cause of this unique phenomenon [32]. The Plant-based diet pattern derived for CCS was a combination of the Vegetable/fruit and Grains pattern in the FFQVP. This pattern is comparable to the Prudent and/or Vegetable/fruit patterns described in other studies, with a high consumption of vegetables, fruits, and other plant-based foods [26, 33, 34], and has been reported to have a protective effect against coronary heart disease [35], type 2 diabetes [26] and CRC [36]. Also, the main food items of whole grain, cereals and grains from the Grains pattern can contain substantial sources of dietary fibre, consumption of which has been shown to be beneficial to health, especially by decreasing the risk of chronic diseases such as CRC [25, 37, 38].

According to findings obtained from the FFQVP and CCS, we conclude that dietary patterns derived by exploratory common factor analysis for those two studies are almost the same, except for the number of factors retained and total variance explained by the retained patterns. These minor differences may be attributed to several reasons. First of all, the sample size may be too small to be representative of the whole population as there were only 554 study participants from the CCS and 192 from the FFQVP. Secondly, distributions of sex and age between the two study populations were significantly different. There were more males in the CCS than in the FFQVP. According to previous studies, dietary patterns are likely to vary between genders as well as age groups. For example, an association between women and higher loadings on healthy dietary patterns has been reported by previous studies [30, 33, 39]. Also, according to one of our earlier studies, older people are more likely to follow a healthier dietary pattern according to results from multivariable linear regression [40]. However, small sample size (stratified by sex or age groups) limited us to conduct factor analysis in this study. Additionally, study participants from the CCS were controls to the CRC cases, and therefore likely to be family members of the cases or diagnosed persons and thus interested in cancer and/or its possible association with nutritional factors. Such individuals may not be able to truly represent the general population. However, study participants in the FFQVP were randomly recruited from the general population. Further, information bias may exist because study participants were required to recall their dietary intakes one or two years prior to the interview or survey.

This study is the first nutritional epidemiological research conducted in the NL population to compare major dietary patterns derived from two independent studies using an identical method but conducted nearly a decade apart. The results of this study provide an overall picture of the dietary exposure of the NL adult population and updated information on the current dietary habits of residents of the province. In addition, this study will provide guidance and reference for future researchers to conduct related studies on this topic through an improved method and study design.

## Conclusion

After a comparison was made of the dietary patterns followed by participants of two separate studies conducted at two different times (FFQVP and the CCS projects), no considerable differences were found. Therefore we conclude that the major dietary patterns followed by the NL adult population have been reasonably stable for almost a decade. However, because of issues on methodology and study design, further investigations to determine the reproducibility and validity of the dietary pattern analysis assessed by the FFQs should be conducted in the future.

## Abbreviations

BTS: Bartlett’s Test of Sphericity
CNF: Canadian Nutrient File
CRC: Colorectal cancer
CSS: Case–control study
ESHA: Elizabeth Stewart Hands and Associations
FFQ: Food-frequency Questionnaire
FFQVP: Food-frequency questionnaire validation project
KMO: Kaiser-Meyer-Olkin
NFCCR: Newfoundland Familial Colorectal Cancer Registry
NL: Newfoundland and Labrador
ON: Ontario
PHQ: Personal history questionnaire
SAS: Statistical Analysis System
